# Implementation of a Quality Improvement Roadmap in the Department of Internal Medicine of an Academic Medical Centre in Singapore

**DOI:** 10.7759/cureus.14877

**Published:** 2021-05-06

**Authors:** Boon Kiat Gary Ong, Tharmmambal Balakrishnan, Mei Ling Kang

**Affiliations:** 1 Medicine, Singapore General Hospital, Singapore, SGP; 2 Internal Medicine, Singapore General Hospital, Singapore, SGP

**Keywords:** quality improvement, management, organisational culture, medical education, leadership

## Abstract

Background: Clarity in directions and constant engagement efforts are crucial to implementing high-quality interventions in Quality Improvement (QI) initiatives. It underpins the success to achieve impactful improvement, effectiveness of interventions through clinical leadership and project sustainability. Our objective was to implement a QI roadmap to improve QI participation of specialists and to clearly align projects and stakeholders to achieve departmental priorities and goals.

Methods: Baseline measurement of Department of Internal Medicine (DIM) specialists involved in QI projects was performed. Root cause analysis and prioritization was conducted to determine the interventions. Series of interventions to address challenges faced by stakeholders to ensure congruency of directions that included collective learning sessions, planning of communication, and documenting progress with checkpoint meetings were carried out. A survey was conducted before and after interventions.

Results: QI projects' participation rates of DIM specialists increased to 82.6% from 26.3% with an increase in uptake in leadership roles from three to nine specialists within the 12 months. The perception survey showed a positive shift in attitudes with greater ease in applying QI tools and concepts, with an increase of 25.7% in 2020 as compared to 2018. With the ease of completing QI projects, DIM specialists became more confident after intervention at 63.2% compared to 42.1% before and also regarded the department to be much stronger in QI culture with an improvement of 51.2%. DIM QI strategic themes model was borne from developing the core focus areas of the departments in order to align existing and prospective QI projects to the established themes.

Conclusion: Department-specific goals and priorities with dedicated interventions are important in driving the interest and ownership to initiate QI projects that align to solve operational problems. The ease in creating the strategic themes model targeting key performance indicators and matching QI projects to the relevant themes, lowers activation barrier and promotes spread due to its simplicity to create and use for communication.

## Introduction

The benefits of applying Quality Improvement (QI) principles in clinical practice have been widely reported with significant improvements to patient care aspects such as care coordination, safety, operational efficiency and access to care, in order to deliver the best care and value to the patient [1]. Furthermore, QI provides a methodological approach towards a problem by understanding the root cause and developing impactful interventions that are targeted at the key problems [2,3].

There is no universal solution for health care delivery organizations seeking to develop and implement effective educational strategies and plans focusing on quality improvement [4]. Team composition and background knowledge knowing the importance, methods, leadership and advocacy are crucial ingredients for project success [5]. In addition to that, participation with perseverance and good management skills for collaboration is essential part of quality improvement [6].

In the Department of Internal Medicine (DIM) at Singapore General Hospital (SGH), being a major tertiary teaching hospital, Patient Care Model and Education are the twin factors that pose challenges to the daily operations of the department. The high clinical workload, delayed discharge processes due to fragmentation of care and patients’ poor understanding of their conditions, contributed to the perpetual challenge of balancing service and residency training needs.

Despite the clear benefits of QI, it is not widely applied and staff of DIM are not deeply involved in QI projects. To further compound the matter, DIM is the largest department in SGH, a tertiary hospital with about 330 inflight patients receiving daily acute care treatment. The number of beds in service, manpower (specialists, various levels of trainees and non-trainees, fresh graduates and students) and the extensive reach of DIM’s footprint brings along many different clinical and operational challenges on the ground. There was a need to prioritise these problems and develop sustainable strategies to counter them in the long-term.

Traditionally, QI projects were done and implemented at DIM wards and clinics but there were key deficiencies and some did not meet the department goals of patient care and safety. These projects were usually led by residents which were not on a sustainable basis as most projects would be abandoned after initial PDSA (Plan, Do, Study, Act) cycle, lacked clinical relevance and improvement efforts were diffused without targeting the root of the problem. Lack of clinical leadership and engagement in the residents’ QI project teams and rushing to complete a QI project within the two-year Advanced Internal Medicine (AIM) Residency programme were common issues raised.

Appropriate measures such as engagement, empowerment and avoiding conflicts must be planned to address challenges faced by stakeholders at various level [7]. Focus of the Head of Department (HOD) who has administrative obligations, specialists with clinical duty demand and trainees with educational requirement needs to be aligned. A QI roadmap was required to clearly guide projects and stakeholders in aligning and achieving departmental priorities and goals. A project team was setup to improve the QI project participation rates from 26.3% to above 80.0% among DIM specialists. The team will be creating interventions that are focused on solving real problems, educating the department on QI process to ease barriers when doing QI projects and ensuring residents to have a good experience in doing a QI project.

## Materials and methods

For this project, quantitative and qualitative data were incorporated as part of the outcomes measurements. Aspects relating to perception surveys, number of DIM QI projects, number of DIM specialists involved in the QI projects and number of DIM specialists in project leadership roles would be the primary measures to assess the effectiveness of the interventions.

At baseline, only five out of 19 DIM specialists were involved in QI projects, of which one DIM specialist assumed the project team leader role. A perception survey using 5-point Likert scale with qualitative feedback was conducted to better understand the context of study and to yield comments from the DIM doctors to assess the QI climate of the department [8]. Upon analyzing the survey results, there were key recurring themes from the respondents, which were lack of understanding about QI, unable to apply QI concepts to daily work, lack of clear departmental priorities and lack of resources (time and advisor). This is aligned to Mittman’s findings, relating to the contextual and technical elements of implementing successful QI projects that showed, training, education and communications would be crucial to increase the participation rates of QI projects and QI engagement levels amongst the DIM doctors [9,10].

The project aimed to be completed within 12 months (Jun 2018 to May 2019). It also aims to collaborate with institution level QI trainers - Process Transformation & Improvement (PTI) Unit to inject QI curriculum into DIM teaching sessions, as well as working closely with DIM HOD and QI lead in the department to develop and implement feasible interventions. 

Design

Focusing on lowering barriers and improving QI education to the specialists were crucial as guiding principles for the team to develop interventions. The attributes considered in developing the solutions included the discussion with HOD and Department’s QI lead to identify and prioritise a list of strategic themes of high clinical relevance to the department. This led to the creation of the department’s QI strategic themes model to align the doctors to the department priorities and focus efforts on the key clinical issues.

Secondly, communicating the priorities was essential to ensure all department specialists were aligned to the agreed priorities and concurrently, the project objectives, timelines and appointed team leaders were established at the same setting. 

Thirdly, it was to involve our institution-level QI faculty by sharing the department’s QI engagement plans to set common grounds together and appoint a designated QI faculty to be the master coach of all registered project teams in order to string all QI project teams’ progress in a coordinated fashion, in order to keep the momentum going.

Fourthly, by working with the institution-level QI faculty, HOD and department QI lead, a mass scoping session was organised to educate specialists on steps to effectively develop mission statement and measures, batch registration of projects to confer legitimacy to projects and accountability of progress by the team leaders. A surveillance mechanism through initiating checkpoint meetings to track status of projects would be required. 

Lastly, QI topics were deliberately planned by QI lead after assessing feedback from project teams and subsequently injected into departmental level teaching sessions so that DIM specialists could be exposed to QI concepts regularly, in order to be more attuned to QI and improve their effectiveness during planning and implementing of QI projects.

Strategy and intervention

Table 1 summarises the intended solution for the key issues and validation to support the solution

**Table 1:** Solution and validation of interventions QI: Quality Improvement; HOD: Head of Department.

Solution	Validation
Theme formation	To streamline the focus in a busy clinical environment with competing education needs based on the need prioritise themes and align projects
HOD engagement	Development of strategic themes and endorsement of QI projects relevant to department themes
Partnership with Experts	Collaborated with institution’s QI experts to develop an approach to engage and impart important QI thought models and skills in implementing a QI project.
Department teaching and knowledge building	Engagement by department champion, through IHI lessons and sharing of QI methodology
Mass scoping session	Institution QI experts led scoping sessions twice at department meetings to guide, critique and collectively teach about good mission statement and developing good measures for project
Monitoring and surveillance	Department QI lead will organise sessions for QI project teams to present their project progress on basis of accountability and reporting

The first intervention (I-1) was to brainstorm and prioritise a list of strategic themes that was relevant to the department, hence the crucial ingredient would be to engage HOD and obtain her buy-in. This PDSA cycle involved creating a list of sub-themes by referencing to the institution and department’s Key Performance Indicators (KPIs). Potential QI project ideas were then brainstormed to find ways to achieve the stipulated targets within preferred time and relevance in alignment with the sub-themes [11]. Projects arising from the brainstorming exercise must contribute directly to address real challenges, like to reduce Average Length of Stay (ALOS) for patients, initiating a multi-disciplinary round to facilitate care management or optimise the clinical care pathway to reduce waste as part of value-driven care. These projects must be matched with relevant KPIs, aligned to both departmental and institutional quality goals.

The second intervention (I-2) included the scheduling of a department meeting session for the HOD. The aim was to address the department on the need to embark on this QI journey by sharing the QI focus areas and formation of QI project teams to manage the problems faced by the department. This meeting was attended by Trainees, Specialists and Nurses, whom were DIM stakeholders. DIM specialists were assigned as either the Project Sponsor or Team Leader to take ownership and ensure sustainability [11,12]. This was a crucial step to demonstrate that all QI projects were endorsed by HOD and the QI lead would assume the surveillance role on top of the assigned QI coach per team.

The third intervention (I-3) was to develop a structured approach for the project teams to learn and apply QI concepts concurrently [12]. In this PDSA cycle, mass QI scoping sessions led by PTI trainers were organised to help project teams frame the mission better and guide teams to implement changes in their projects. It was essential to teach the members how to address the problems with a QI lens and the methodology to develop solutions through root cause(s) identification. This was followed by two-monthly meetings for individual project updates in order to continue engagement and support from everyone to complete projects.

The fourth intervention (I-4) was to formalise the registration of QI project in the institution and officialise the appointment of the project team’s sponsor, leader and members. This administrative ceremony was important to establish a psychological compact between the members and institution to ensure commitment to project implementation and success. Institution QI trainers would be assigned to registered project teams as QI coaches to provide guidance on QI methodology and answer any project queries, usually on success measures and data collation.

The fifth intervention (I-5) was the convening of a checkpoint meeting for all project teams as part of surveillance to ensure progress and accountability for project. Concurrently, the meeting served as a learning platform for all the project teams to gain exposure to other projects, interventions and sharing by team members on implementation challenges. Furthermore, this would be an excellent impetus for project teams to report on progress.

The sixth intervention (I-6) consisted of a formative approach to galvanise the department to proactively learn about QI concepts [13]. Screening of Institute of Healthcare Improvement (IHI) videos and attempting quizzes in a single setting to ensure that there was an element of collective learning and building a culture to speak the same QI language. This was encouraged so that concepts or examples learned from the screening could be actively applied for opportunistic learning. Proactively teaching the QI tools learnt and sharing of the application reduced fear of performing QI. Such sessions were actively incorporated into the formal meetings and teaching sessions and counted as Continuing Medical Education (CME) points.

## Results

**Figure 1:**
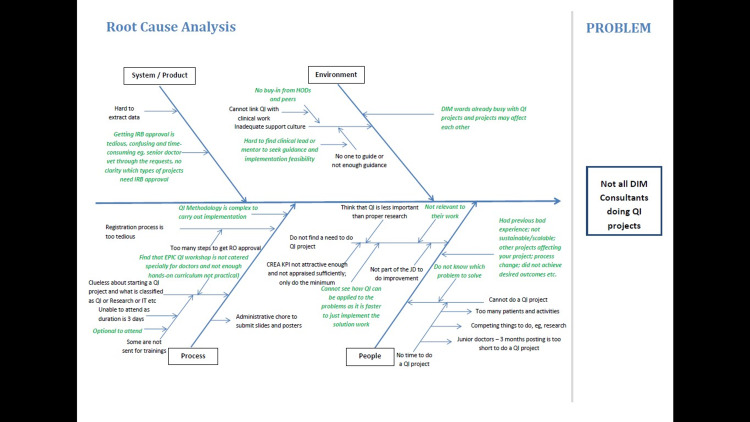
Ishikawa diagram showing the reasons for consultants not participating in quality improvement (QI) projects Green text - problem to target intervention through prioritization matrix.

**Figure 2:**
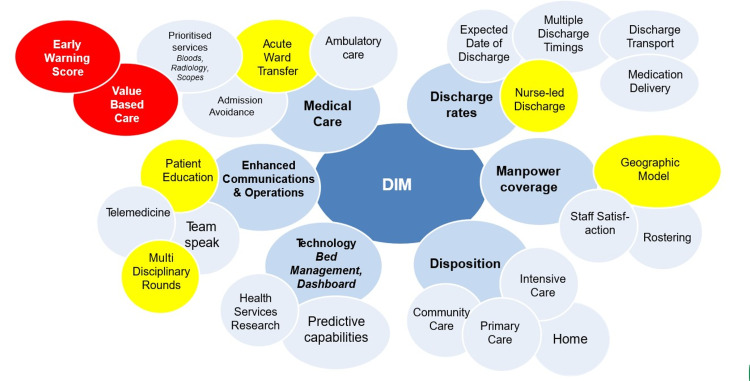
Quality improvement (QI) strategic themes model illustrating the department’s priorities red - first priority, yellow - second priority, pale blue - last priority, light blue - subthemes.

Theme formation focused on main areas of processes and structure targeting KPI, patient education, safety-based, according to the Ishikawa diagram (Figure 1). In addition to the improved perception of the interventions, the team developed a QI management model to ensure clarity on department priorities (Figure 2). The next step was to establish relevant levers or indicators that would contribute to the success of the listed priority e.g., optimising ward operations would entail reducing ALOS in order to bring create bed savings for more acute cases to be admitted. Subsequently, ALOS reduction projects could be targeted at various diagnoses due to specific nature of disease management. With each disease-specific ALOS reduction project being registered, the project team would then employ rapid PDSA cycles to improve ways in their clinical and operational processes to reduce ALOS accordingly. Other strategic themes could replicate the same process so that the department would have an oversight of all priorities with corresponding projects targeting respective projects reflected on a single diagram (Figure 1).

Measurements were taken at quarterly intervals against the baseline, over a period of 12 months. At baseline, only 26.3% (five out of 19) of specialists were involved in projects and only three out of the five involved specialists assumed team leadership roles (Project Sponsor and Team Leader). After the 12-month project duration, there were six registered DIM projects. The number of specialists involved in QI projects increased significantly to 82.6% (19 out of 23), with nine out of the 19 involved specialists assumed leadership roles.

At post-intervention stage, there was a general positive shift in the QI perception by DIM specialists. Firstly, the DIM specialists showed greater ease in applying QI tools and concepts, with an improvement of 25.7%, when compared between Year 2020 (36.8%) and Year 2018 (11.1%). In terms of ease of completing QI projects, DIM specialists were more confident in this aspect, showing an improvement of 21.1% from Year 2018 (42.1%) to Year 2020 (63.2%). DIM specialist regarded the department to be much stronger in QI culture with an improvement of 51.2%, from Year 2018 (22.5%) to Year 2020 (73.7%). Refer to Table 2 for the collated survey responses.

**Table 2:** Survey responses using 5-point Likert Scale (1=Strongly Disagree; 5=Strongly Agree) DIM: Department of Internal Medicine; QI: Quality Improvement.

Survey response	2018		2020		P–Value for score ≥ 4
	n (%) scored <4	n (%) scored ≥ 4	n (%) scored <4	n (%) scored ≥ 4	
It is easy for me to complete a QI project	8 (88.9%)	1 (11.1%)	12 (63.2%)	7 (36.8%)	0.21
I am able to easily apply QI Tools and Concepts	11 (57.9%)	8 (42.1%)	7 (36.8%)	12 (63.2%)	0.29
DIM has a strong QI culture among the doctors	7 (77.8%)	2 (22.5%)	5 (26.3%)	14 (73.7%)	0.02

The roll-out of the strategic themes, structured programme to allow doctors to have the full QI experience and collective learning have shown glowing results, especially in the domains of QI knowledge and environment. Feedback from the ground was generally positive and the trainee doctors, whom were relatively new to the system, benefited greatly with clarity in clinical guidance from the team leader and able to solve true departmental problems were important in the entire QI project experience. From the department’s perspective, it is important that specialist-led projects could be more sustainable in the long-run, have true problems to be solved and able to instill the improvement mindset in trainees to develop them into better clinicians in the future.

Qualitative responses were gathered after the implementation of interventions, with a mix of both positive and negative feedback (Table 3). It was inferred that the positive responses were largely from the converted lot which believed that QI formed an important element in improving clinical processes and hinged mainly on the availability of resources such as QI coach, good teamwork, active participation, and constructive collaboration with other healthcare roles like nursing.

**Table 3:** Qualitative feedback by survey respondents QI: Quality Improvement.

Positive Feedback
“Members were participative, good tips and guidance from QI mentor (coach).”
“It was about heart failure education and went very well with one main leader.”
“Clearly defined the focus and priority of projects.”
“The QI projects I had been involved thus far has brought much inter-professional education value as it involves many different types of healthcare workers”
“Overall, my QI project was conducted smoothly and all team members contributed a lot. We had a lot of reviews and support, which made the design and implementation of our QI project much easier to do. I think we can build more future QI projects based on our current foundation.”
Negative Feedback
“Mandatory QI project completion for residency is one way to try to get everyone familiar with the QI methodology. But the spirit of QI might be lost as a result.”
“The longer the duration of project, the lesser the enthusiasm of team members. Only very few members continue to keep the project going.”
“Concerned about competing interests of various QI in already busy wards.”
“Difficulty lies in defining the scope of QI project. Too big to be realistic within a certain amount of time or too small to be impactful.”

## Discussion

In a busy clinical environment, it is crucial to be focused on the priority and strategy in order to achieve the target. In our study, the formation of the strategic themes model was crucial in guiding the department in the right direction [14]. The key to improvement was prioritising the department’s key strategic themes, initiating QI project ideas, involving a multi-disciplinary team in tackling immediate problems, and incorporating QI as part of the teaching curriculum for the department.

In the course of the project, active HOD participation was the turning point to improve DIM doctors’ engagement in QI. Complementing these efforts, the involvement of the institution’s QI expert added weight to DIM’s QI journey [15]. Another reason crucial element was role-modelling. The HOD and department QI lead became the main QI evangelist assuming the role as a knowledge centre for all doctors to enquire about QI such as scoping of problems, QI methodology, and QI administration matters [16]. Progressively, the DIM doctors became more receptive and attuned to QI and they started to work closely with the project team and applied QI lingo in the conversations. This trend demonstrated the efficacy of having influencers to drive the change and also a supportive environment to nurture the spirit of QI.

As the department’s specialists increasingly assumed roles of project sponsors or team leaders and residents as team members. This phenomenon becomes a strategic move because the specialist will be held accountable for the project progress, ensure improvement cycles were made sustainable with a better appreciation of the problems, and guide residents would be better guided in the project, hence addressing the leadership and clinical relevance issues [17]. This yielded mutual benefits with the specialists having a better shot at succeeding or sustaining projects; trainees were able to have clearer directions and better experiential learning in a QI project. 

An important component in building up a QI culture depended heavily on the continuous engagement with the department specialists. It was done through regular scheduling of QI-related education sessions to build up domain knowledge, followed by a series of collective discussion sessions as part of the discourse and shared responsibility, and as a department entity to overcome any implementation challenges [18]. This practice helped to cement the QI knowledge and methodology. The reflective element reinforced the foundational knowledge and enhanced the learning experience in the domain of QI.

The current approach of having “ready-made” projects might bring about the possible stifling effects which limited the creativity and proactive spirit of residents in coming up ground-up projects. The team examined this matter and looked at historical evidence. We believe this approach would yield better outcomes when projects were made available to residents. It was important to note that this approach still allows a channel for residents to float up new project ideas and at the same time, having the majority of clinically relevant projects driven by specialists.

## Conclusions

This project was initiated based on the main objective of addressing emerging challenges faced by the department through employing QI methodology to achieve this objective. The doctors were surveyed to gain a better understanding of the barriers toward QI and a plethora of responses were garnered and stratified into major categories for the team to tackle. The key success determinants included the complete involvement of HOD, development of the strategic themes, specialist-led projects for sustainability, and clinical relevance. With these, the HOD would gain better oversight of the department QI projects, empowered specialists to own and account the progress of the projects; From the education perspective, the residents would have a better learning experience from the QI project with clear leadership by the project sponsor and team leader and ultimately, able to meet the programme exit requirements. From this experience, the team planned to up the ante by looking into developing new project ideas with better quality and impact as the department moved towards the next phase in this QI journey. The DIM specialists were getting better at approaching daily work problems with QI lens and to see problems from a systems-level point of view. Solutions development by the doctors were gradually becoming holistic by considering upstream, downstream and stakeholder (e.g., nurses, pharmacists, administrators) impacts too. Hence, the implemented interventions were clearly simple steps that could be easily implemented. The outcomes were very impactful which could percolate from department to organisation and most importantly, benefit patient care and improve staff engagement. We aim to improve on the model and also to address some of the comments from survey responders in the future.
